# Percutaneous Vertebral Reconstruction (PVR) Technique of Pathological Compression Fractures: An Innovative Combined Treatment of Microwave Ablation, Bilateral Expandable Titanium SpineJack Implants Followed by Vertebroplasty

**DOI:** 10.3390/jcm12134178

**Published:** 2023-06-21

**Authors:** Claudio Pusceddu, Salvatore Marsico, Daniele Derudas, Nicola Ballicu, Luca Melis, Stefano Zedda, Carlo de Felice, Alessandro Calabrese, Davide De Francesco, Massimo Venturini, Domiziana Santucci, Eliodoro Faiella

**Affiliations:** 1 Department of Oncological and Interventional Radiology, Businco Hospital, 09121 Cagliari, Italy; clapusceddu@gmail.com (C.P.); nicola.ballicu@aob.it (N.B.); stefanozedda1@gmail.com (S.Z.); 2 Department of Radiology, Hospital del Mar, 08003 Barcelona, Spain; salvatore.marsico@hotmail.it; 3 Department of Hematology, Businco Hospital, 09121 Cagliari, Italy; derudas74@gmail.com; 4 Nuclear Medicine Department, Businco Hospital, 09121 Cagliari, Italy; doclucamelis@tiscali.it; 5 Department of Radiological Sciences, Oncology and Pathology, Umberto I Hospital, Sapienza University of Rome, Viale del Policlinico, 105, 00161 Rome, Italy; c.df@uniroma1.it (C.d.F.); alessandro.calabrese@uniroma1.it (A.C.); 6 Department of Anesthesiology, Perioperative and Pain Medicine, Stanford University School of Medicine, 300 Pasteur Drive, Stanford, CA 94305, USA; ddefra@stanford.edu; 7 Diagnostic and Interventional Radiology Department, Circolo Hospital, ASST Sette Laghi, 21100 Varese, Italy; massimo.venturini@uninsubria.it; 8 Department of Medicine and Surgery, Insubria University, 21100 Varese, Italy; 9 Department of Radiology, Fondazione Policlinico Universitario Campus Bio-Medico, Via Alvaro del Portillo, 200, 00128 Roma, Italy; d.santucci@policlinicocampus.it

**Keywords:** spine metastases, microwave ablation, percutaneous therapies, expandable titanium SpineJack implants, interventional radiology

## Abstract

(1) Background: to retrospectively evaluate safety and efficacy of combined microwave ablation (MWA) and bilateral expandable titanium SpineJack (SJ) implants followed by vertebroplasty (VP) for the treatment of painful thoracolumbar pathological vertebral compression fracture. (2) Methods: from July 2017 to October 2022, twenty-eight patients (13 women and 15 men; mean age 68 ± 11 years) with a history of primary neoplasm and thirty-six painful vertebral metastases with vertebral compression fracture underwent combined MWA and bilateral expandable titanium SpineJack implants with vertebroplasty. We analyzed safety through complications rate, and efficacy through vertebral height restoration and pain decrease, evaluated using a visual analogue scale (VAS), and Functional Mobility Scale (FMS), and local tumor control. Contrast-enhanced CT scans were performed at 1, 3, and 6 months and a contrast-enhanced spine MRI at 6 months after the procedure. (3) Results: Technical success rate was 100%. No procedure-related major complications or death occurred. Vertebral height restoration was observed in 22 levels (58%), with a mean anterior height restoration of 2.6 mm ± 0.6 and a mean middle height restoration of 4.4 mm ± 0.6 (*p* < 0.001). Mean VAS score of pain evaluation on the day before treatment was 6.3 ± 1.5 (range 4–9). At the 6-month evaluation, the median VAS score for pain was 0.4 ± 0.6 (range 0–2) with a mean reduction of 93.65% (6.8 ± 0.7 vs. 0.4 ± 0.6; *p* < 0.000) compared with baseline evaluation. Contrast-enhanced CT scans were performed at 1, 3, and 6 months and a contrast-enhanced spine MRI was performed at 6 months after the procedure, showing no local recurrence, implant displacement, or new fractures in the treated site. (4) Conclusions: combined microwave ablation and bilateral expandable titanium SpineJack implants with vertebroplasty is a safe and effective procedure for the treatment of pathological compressive vertebral fractures. The vertebral stabilization achieved early and persistent pain relief, increasing patient mobility, improving recovery of walking capacity, and providing local tumor control.

## 1. Introduction

Bone metastases are a markedly widespread and common nosological entity in the context of oncological diseases.

The treatment of vertebral metastases is very complex due to the presence of severe pain associated with instability and consequent neurological deficits that determines an increase in morbidity and mortality.

Conventional therapies, such as chemotherapy, radiotherapy, hormone therapy, bisphosphonates, and analgesics, are effective in controlling pain and instability in most cases, but due to the increased incidence of spinal metastases, non-response to conventional treatments or contraindications to them, the role of interventional radiology in this field has been increased [1,2].

Main interventional radiology techniques, such as radiofrequency (RF), alcohol, interstitial laser (IL), microwaves, and cryoablation (CA), are gaining an increasingly important role in the local treatment, generally with palliative intent, of vertebral metastases, although in some patients it can also be performed to achieve local tumor debulking and spine stabilization [3,4,5,6,7,8,9,10,11,12].

CIRSE (Cardiovascular and Interventional Radiology Society of Europe) guidelines indicate that vertebral augmentation techniques (VAPs) such as vertebroplasty (VPT), kyphoplasty (KPT), and vertebral implants (VI), also combined with focal ablation modalities, can be usefully and successfully combined to prevent or stabilize pathological fractures in painful vertebrae with extensive osteolysis that do not respond to conventional therapies or in combination with them [13].

Only one study in the literature explores the possibility of using SpineJack (SJ) for the treatment of vertebral fractures secondary to metastatic tumor infiltration, showing promising results [14].

The purpose of our study is to describe an innovative percutaneous combined treatment composed of microwave ablation (MWA) with bilateral expandable titanium SpineJack implants followed by vertebroplasty and to evaluate its safety and efficacy for the management of refractory painful vertebral metastases, assessing pain reduction, and vertebral structural restoring.

## 2. Materials and Methods

This study was approved by the ethics committee of our institution. Informed consent was obtained from all individual participants included in the study.

This is a single-center retrospective study including 28 patients (13 women and 15 men; mean age: 64 years, range 41–80 years) with a history of primary neoplasm (8 breast carcinoma, 5 non-small cell lung cancer, 2 kidney, 2 thyroid, 2 oral cavity, 2 pancreas, 2 bladder, 1 testicle, 1 NET (neuro-endocrine tumor), 1 colon, 1 parotid, and 1 melanoma) with 36 vertebral metastases underwent combined MWA with bilateral expandable titanium SpineJack implants followed by vertebroplasty (Table 1).

The inclusion criteria were the presence of vertebral metastasis determining a vertebral body stress fracture detected on computed tomography (CT) or magnetic resonance imaging (MRI) scan, in patients with life expectancy greater than 2 months.

The exclusion criteria were the presence of a primary spinal tumor, complete vertebral collapse, extensive epidural and spinal canal infiltration (more than a third of the extension of the circumference of the epidural space), severe vertebral canal stenosis, and moderate-severe neurologic deficits.

The pre-operative evaluation consisted of a combined oncological-radiological interventional clinical examination, evaluating the severity of pain measured using the visual analog scale (VAS) and Functional Mobility Scale (FMS).

The VAS score was subsequently evaluated at 1 week and 1, 3, and 6 months follow-up. The FMS was recorded 1 month after the treatment to assess the effect of the treatment on mobility and the ability to walk. A 4-point FMS classification was used: 4, bedridden; 3, use of a wheelchair; 2, limited painful ambulation; 1, normal ambulation.

All patients performed a pre-procedural contrast-enhanced CT scan to assess the location, size, and radiological characteristics of the lesion and to define the procedural planning. The combined procedure was performed in a single vertebra in 20 patients, in 2 vertebrae in 5 patients, and in 3 vertebrae in 1 patient. In 2 patients, the procedure consisted of SJ implants and vertebroplasty without ablation because they had already been treated with MWA (Table 1). Control contrast-enhanced CT scans were acquired 3 and 6 months after the procedure according to routine oncologic follow-up.

Drug therapy (NSAIDs and opioids) was discontinued 1 week after the treatment and resumed in cases of persistence or exacerbation of painful symptoms. Antibiotic prophylaxis was administered 30 min before the start of the procedure with a single dose of 2 mg of i.v. cefazolin.

The combined treatment of MWA and expandable titanium SpineJack implants was performed under dual CT guidance and fluoroscopy guidance to allow the correct positioning of the antenna, evaluate the ablation area, obtain a correct visualization during advancement and expansion of the SJ implants, control potential posterior wall protrusion and monitor any leaks during cement injection.

CT acquisition data were: 5 mm collimation at 80–140 mA (TC system: SOMATOM Sensation, Siemens, AG, Forchheim, Germany). Patients, in a prone position, underwent conscious sedation with continuous intravenous infusion of fentanyl citrate (0.1 mg/2 mL diluted 1:10 with saline) and received local anesthesia with subcutaneous injection of lidocaine hydrochloride at 2%.

After periosteum anesthesia with bupivacaine hydrochloride at 0.9% using 18 Gauge Chiba needle, two 10 Gauge beveled bone needles (Thiebaud Biomedical Devices, Margencel, France) were directly inserted into the posterior aspect of the vertebral body via a bilateral transpedicular approach.

Through this access, the microwave antennas were inserted coaxially for the ablation. Percutaneous MWA was performed using a 2.45 GHz microwave generator (AMICA-GEN, HS Hospital Service, Aprilia, Italy) which supplies energy through a 14-gauge interstitial antenna, mini choked, and water-cooled (AMICA-GEN).

If the metastasis was limited to half of the vertebral body, the lesion was ablated using a single 14-gauge antenna.

If the lesion exceeded the midline of the vertebral body, 2 MWA antennas were positioned with bi-pedicle access.

Once the ablation was finished, the microwave needles were removed and two blunt guidewires (Stryker, Rome, Italy) were inserted coaxially through the same access cannula repositioned in the posterior vertebral third, up to the anterior vertebral third.

A designed drill (Stryker) mounted on a working cannula was gently advanced coaxially into the vertebral body until the desired position of the implant, approximately up to 5 mm from the anterior wall.

After the removal of the first drill, an acrylic plug is left in place until both bone canals have been prepared for implantation. When the drill was then removed, the working cannula was left on site to allow the subsequent introduction of the implants.

After preparation of both sides, the two SpineJacks^®^ (Stryker Corp, Kalamazoo, MI, USA) were inserted into the vertebral body through the working cannulae and were gradually and simultaneously deployed, under fluoroscopic control, by turning the expanders’’ handles clockwise until height restoration and kyphosis reduction were judged satisfactory.

The implants were detached by unscrewing the quick-release pin at the tip of the handles.

Finally, poly-methyl-methacrylate (PMMA) bone cement (SpinePlex^®^ radiopaque bone cement; Stryker Corp, Kalamazoo, MI, USA) was slowly injected under real-time fluoroscopy through the same working cannula into the vertebral body and around the devices until the optimal filling was obtained. Following the injection of cement, the working cannulas were extracted.

**Figure 1:**
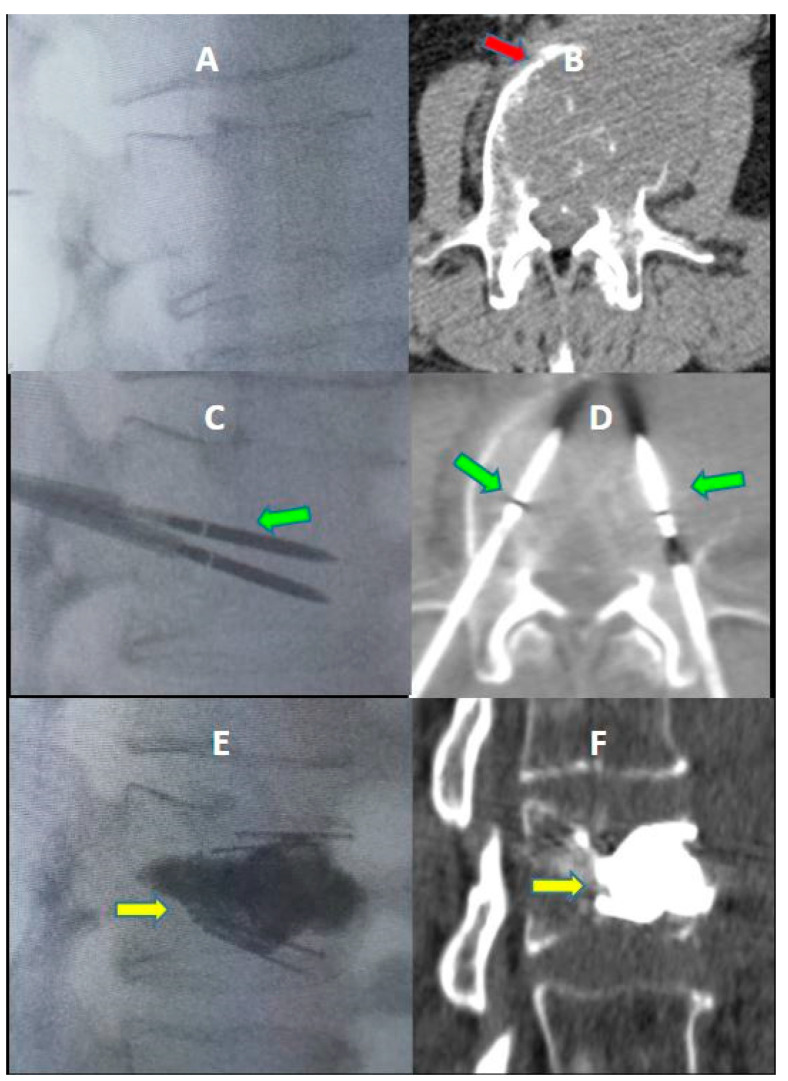
A 58-year-old male with a history of colon cancer with lumbar pain. (**A**,**B**) lateral fluoroscopic image—Axial CT image showed a pathological compression fracture of the superior endplate of the vertebral body of L3 with extension to the periosseous soft tissue (red arrow in (**B**)); (**C**,**D**) lateral fluoroscopic image ©—axial CT image (**D**); microwave antennae inserted coaxially through cannulae for ablation (green arrows); (**E**,**F**) lateral fluoroscopic image (**E**)—axial CT image (**F**); post-procedure control showed a correct expansion of the vertebra with a homogeneous distribution of cement into the vertebral body (yellow arrows). No procedural complications were observed.

An immediate post-procedural no contrast-enhanced CT scan was performed to evaluate the results and any complications (Figure 1).

All patients were followed up for up to six months.

**Figure 2:**
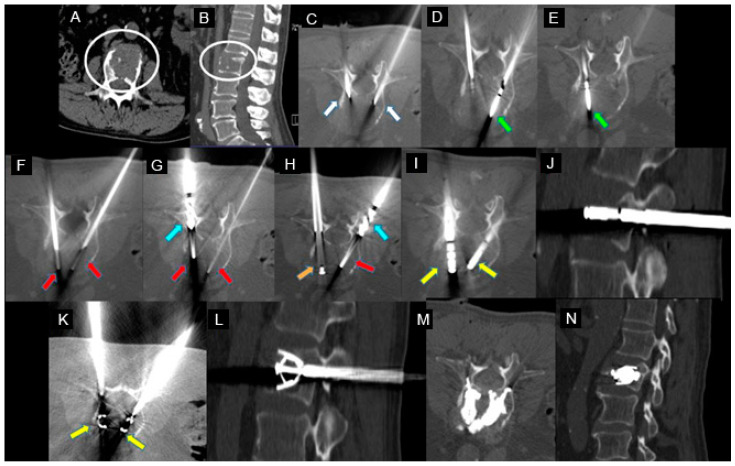
A 73-year-old male with a history of kidney cancer with lumbar pain. (**A**,**B**) Axial (**A**) and sagittal (**B**) CT scan showing metastatic lytic lesion of the L2 vertebra (white circle in (**A**,**B**)). (**C**) Axial CT image; two 10 Gauge beveled bone needles (Thiebaud Biomedical Devices) directly inserted into the posterior aspect of the vertebral body via a bilateral transpedicular approach (white arrows). (**D**) Axial CT image; first (right) microwave antenna inserted coaxially through the cannula for ablation (green arrow). (**E**) Axial CT image; second (left) microwave antenna inserted coaxially through the cannula for ablation (green arrow). (**F**) Axial CT image; after ablation, microwave needles were removed, and two blunt guidewires (red arrows) were inserted coaxially through the same access cannula. (**G**): Axial CT image; a designed drill (blue arrow) mounted on a working cannula was gently advanced coaxially into the left vertebral body until the desired position of the implant. Blunt guidewires (red arrows). (**H**) Axial CT image: after the removal of the left drill, an acrylic plug is left in place (orange arrow). Advancement of the drill to the right (blue arrow). Blunt guidewire (red arrow). (**I**–**L**): Axial CT image; after preparation of both sides, two SpineJack^®^ (yellow arrows) were inserted into the vertebral body through the working cannulae (**I**,**J**) and were gradually and simultaneously deployed (**K**,**L**). (**M**,**N**) Post-procedure control—CT in the axial (**M**) and sagittal (**N**) planes showed a correct expansion of the vertebra with a homogeneous distribution of the vertebral cement. No periprocedural complications were observed.

The details of the spine jack positioning procedure are described in Figure 2.

### Statistical Analysis

For the purposes of this study, continuous variables were reported as mean ± standard deviation (SD). Differences between the average VAS score at 1 week, 1, 3, and 6 months and FMS at 1 month after the procedure was evaluated by means of Student’s t-test or Fisher’s exact test as appropriate. A *p*-value less than 0.05 was taken as significant. Statistical analysis was performed using OpenStat software version 11.9.08.

## 3. Results

A total of 76 expandable titanium SpineJack implants were inserted into 38 vertebrae.

**Figure 3:**
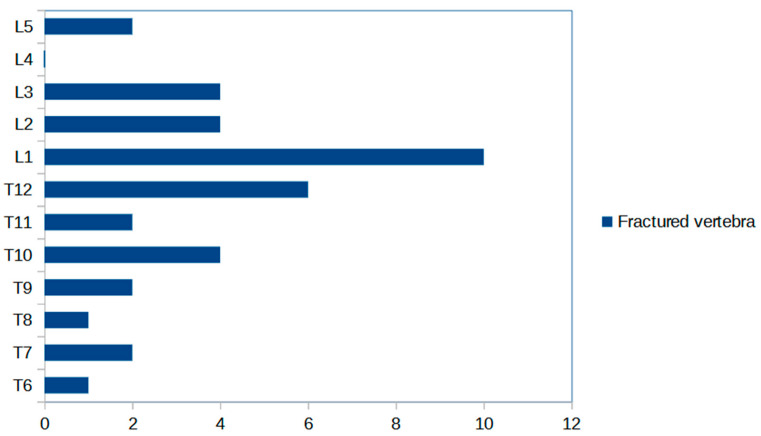
Fracture distribution graph comparison; T = thoracic; L = lumbar.

The levels of the treated vertebrae can be seen in Figure 3.

The technical success rate was 100% (82/82) without major complications. A single MWA antenna was used in 22 sessions, and two antennas were used in 14 sessions.

Minimal leakage of cement occurred in three procedures (8%), one anterolateral, one posterolateral and one intradiscal leakage, without clinical impact.

Two patients developed a secondary vertebral fracture in a caudal segment, respectively, 7 and 10 days after the procedure.

Both adjacent fractures were successfully treated with implantation of SpineJack implants (Patient 4 and 15 Table 1).

**Figure 4:**
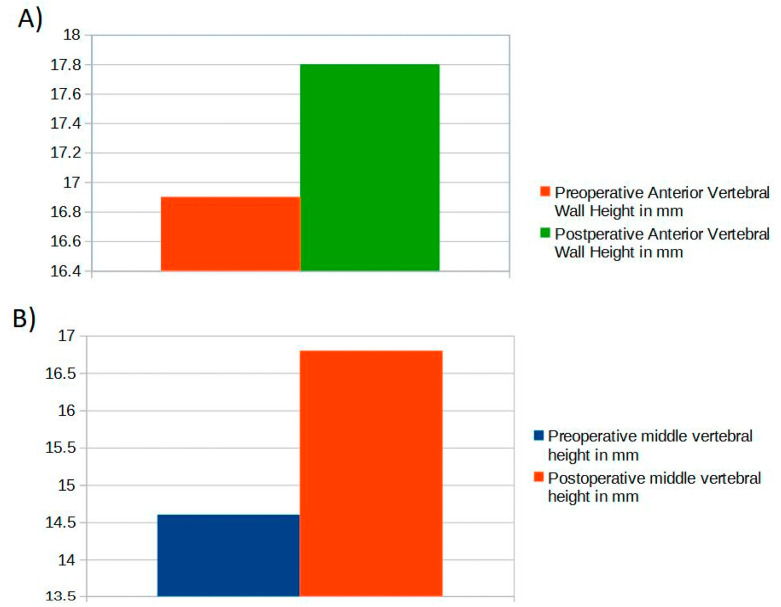
(**A**) Mean pre- and post-operative anterior vertebral height values; (**B**) mean pre- and post-operative medium vertebral height values.

Vertebral height restoration was observed in 22 vertebrae (58%), with a mean anterior column height restoration of 2.6 mm ± 0.6 and a mean middle column height restoration of 4.4 mm ± 0.6 (*p* < 0.001) (Figure 4).

All patients were discharged 24 h after the treatment in stable and uncomplicated conditions.

No patients were lost at the follow-up at 6 months.

The mean VAS score on the day before treatment was 6.3 ± 1.5 (range 4–9).

**Figure 5:**
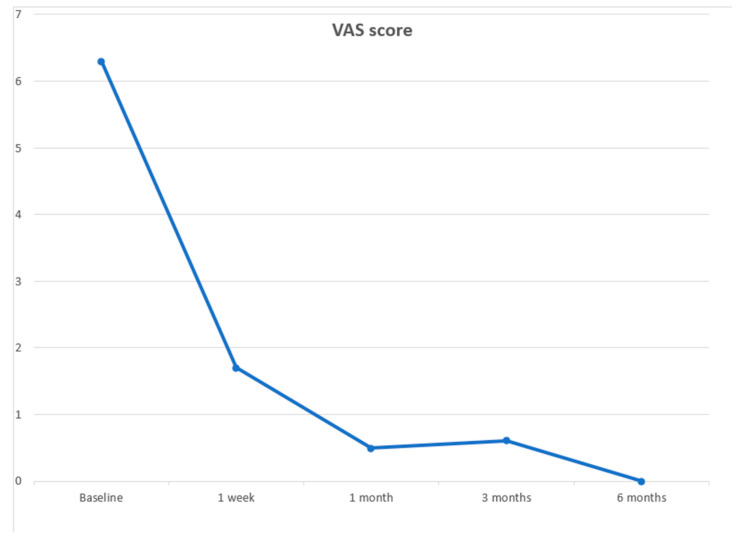
Median VAS (Visual Analogic Scale) score follow-up evaluated before and prospectively after 1 week, and 1, 3, and 6 months from the treatment. The blue line indicates patients’ changes in pain.

One week after treatment, the median VAS score was 1.7 ± 1.2 (range, 0–4) with a mean reduction of 73.02% (6.8 ± 0.7 vs. 1.7 ± 1.2; *p* < 0.000; Figure 5).

One month after treatment, the median VAS score was 0.5 ± 0.6 (range, 0–3) with a mean reduction of 92.06% (6.8 ± 0.7 vs. 0.5 ± 0.6; *p* < 0.000; Figure 5) compared with baseline evaluation.

At the 3-month evaluation, the median VAS score was 0.6 ± 0.6 (range 0–2) with a mean reduction of 90.48% (6.8 ± 0.7 vs. 0.6 ± 0.6; *p* < 0.000; Figure 5) compared with baseline evaluation.

At the 6-month evaluation, the median VAS score was 0.4 ± 0.6 (range 0–2) with a mean reduction of 93.65% (6.8 ± 0.7 vs. 0.4 ± 0.6; *p* < 0.000; Figure 5) compared with baseline evaluation.

The mean FMS on the day before treatment was 2.4 ± 0.4 (range 1–4). One month after treatment, the median FMS of disability was 1.4 ± 0.4 (range, 1–3) with a mean reduction of −41.67% (3.1 ± 0.7 vs. 1.4 ± 0.4; *p* < 0.000) compared with baseline evaluation.

In particular, before the procedure, 2 (7.2%) patients reported normal ambulation on the FMS, 15 (53.5%) limited painful ambulation, 7 (25%) the use of a wheelchair, and 4 (14.3%) were bedridden.

Three of the seven patients who reported the use of a wheelchair before the procedure acquired normal ambulation 1 month after the procedure.

All seven patients who presented limited, painful ambulation before the procedure improved mobility and reported normal ambulation after the procedure.

Of the four bedridden patients, two reported limited painful ambulation after one month, and the other two improved mobility acquiring normal ambulation one month after the procedure. During follow-up, no infectious complications were observed.

**Figure 6:**
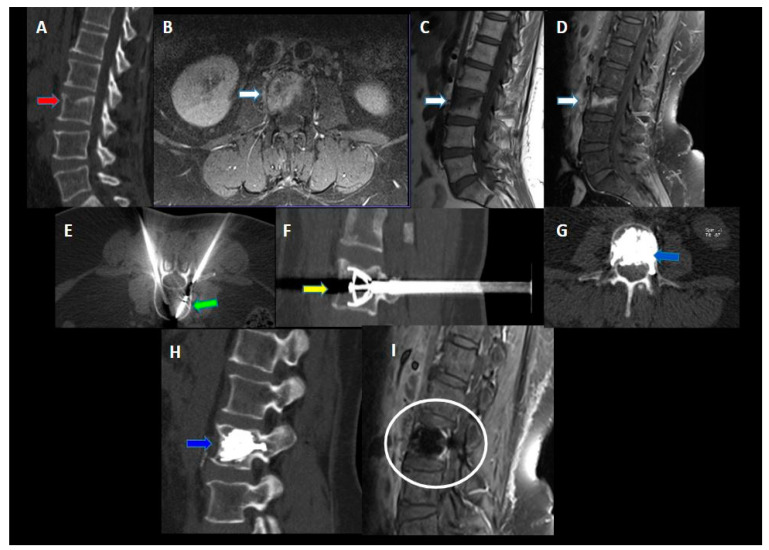
A 50-year-old female with a history of breast cancer with lumbar pain. (**A**) A CT of the lumbar spine in the sagittal plane shows a compression fracture of the superior endplate of the vertebral body of L3 (red arrow). (**B**–**D**) CE-MRI (in (**B**) the axial plane—CE T1 Fat-Sat sequence, in (**C**) the sagittal plane—T1 TSE sequence, and in (**D**) the sagittal plane—sagittal plane—CE T1 Fat-Sat sequence) confirms the presence of active vertebral fracture associated with a hyperintense focal lesion in T1 Fat-Sat sequences (white arrows). (**E**) Axial CT image; right microwave antenna inserted coaxially through the cannula for ablation (green arrow). (**F**) Sagittal MIP (maximum intensity projection) image after the opening of the SJ implants (yellow arrow). (**G**,**H**) Post-procedure control—CT in the axial (**G**) and sagittal (**H**) planes showed a correct expansion of the vertebra and a homogeneous cement filling (blue arrows). No procedural complications were observed. (**I**) follow-up MRI 12 months after the procedure (the sagittal plane-T1 Fat-Sat sequence): no evidence of local recurrence of the disease or complications in the treated vertebra (white circle).

Contrast-enhanced CT scans performed at 1, 3, and 6 months and contrast-enhanced spine MRI performed at 6 months after the procedure showed no local recurrence, implant displacement, or new fractures in the treated site (Figure 6).

## 4. Discussion

Bone metastases, which are frequently localized in the spine, can affect more than half of patients with a history of malignant tumors and are often the cause of pain, instability, and disability, affecting the quality of life.

Given the complexity of metastatic bone pathology and its clinical consequences, a multidisciplinary approach is required.

The most used systemic and focal therapies for the treatment of bone metastases are chemotherapy, radiotherapy, hormone therapy, bisphosphonates, and analgesics [1].

In 2017, Tsoumakidou et al. pointed out in the CIRSE guidelines that vertebra augmentation techniques, such as vertebroplasty, kyphoplasty, and vertebral implants, are indicated in the treatment of vertebral compression fractures.

These treatments are also indicated for painful vertebrae fractures with extensive osteolysis due to malignant infiltration by multiple myeloma, lymphoma, and metastasis that do not respond to conventional therapies or in combination with them [14].

Vertebroplasty, while improving the pain associated with vertebral collapse, does not allow recovery of the vertebral body height and prevent spinal deformity, which can sometimes be associated with new vertebral fractures.

Furthermore, VPT does not allow a suitable action in the local treatment of the tumor and is associated with a moderate rate of cement leaks [15]. Balloon kyphoplasty and other vertebral implants have been developed to further improve the cement deposition and stabilization of cancer-related fractures [16,17]. SpineJack is an expandable intravertebral titanium implant that allows the restoration of vertebral height and the maintenance of a correct kyphotic angle of the spine resulting in a more balanced distribution of the craniocaudal thrust forces on the fractured vertebrae and on the whole spinal column [18]. It is widely used in the treatment of vertebral compression fractures [19,20,21,22,23,24,25].

Follow-up studies directly comparing SpineJack implant to KPT in the treatment of vertebral post-traumatic compression fractures demonstrated that the vertebral height correction obtained with SJ is associated with less spinal deformity; less cement is used than with KPT and the incidence of adjacent fractures is significantly less in SJ (3 to 5%) compared to 15–20% with KPT [26,27].

In 2022, Cornelis et al. published a single-center retrospective review study on 13 patients to evaluate the applicability of SJ, using cone-beam CT guidance, in the treatment of vertebral fractures secondary to tumor infiltration with promising results [16].

Our results are similar in terms of technical success without major complications confirming that it is a feasible and safe procedure.

Unlike that study, all our patients underwent procedures under conscious sedation and local anesthesia, with no need for general anesthesia.

We believe that the use of CT scan in combination with fluoroscopic guidance allows both a correct positioning of the MWA antennae and SJ implants without significant side effects or procedural complications.

The idea of combining ablation treatment with SJ implantation prior to vertebroplasty is based on different scientific assumptions. The 2022 CIRSE guidelines indicate that in load-bearing and long bones, ablation and consolidation can be usefully combined to effect ablation and palliation and prevent or stabilize pathological fractures, and if the goal of the treatment is curative, percutaneous osteoplasty should always be preceded by a definitive ablative treatment [13].

In 2020, guidelines in Oncology (Bone health in cancer: ESMO Clinical Practice Guidelines), the use of combined ablative percutaneous therapies and VAPs in the treatment of spine metastases were suggested to obtain pain relief and tumor burden reduction in bone.

Minimally-invasive ablation techniques and VAPs are used in combination to reduce tumor mass, create a cavity and stabilize the vertebral body [28]. Mohme et al. observed that peripheral circulating tumor cells (CTCs) are significantly increased due to vertebral cement augmentation procedures, justifying the rationale of combined ablation techniques option to reduce the increased release of CTC associated with cementoplasty [29].

A systematic review of the literature by Sagoo et al. confirmed that the use of MWA, associated with vertebroplasty to treat patients with painful vertebral metastases, is capable of achieving both effective pain relief and local disease control, as already evidenced in previous studies [30,31,32,33].

The pathological replacement of a vertebral body is associated both with the phenomena of osteolysis and the cause of pain, and often with structural subversion based on trabecular collapse and extra-vertebral extension.

The purpose of the combined treatment of ablation with SJ implant and vertebroplasty is the structural recovery of the vertebra so as to obtain both an analgesic effect and recovery of the patient’s mobility.

**Figure 7:**
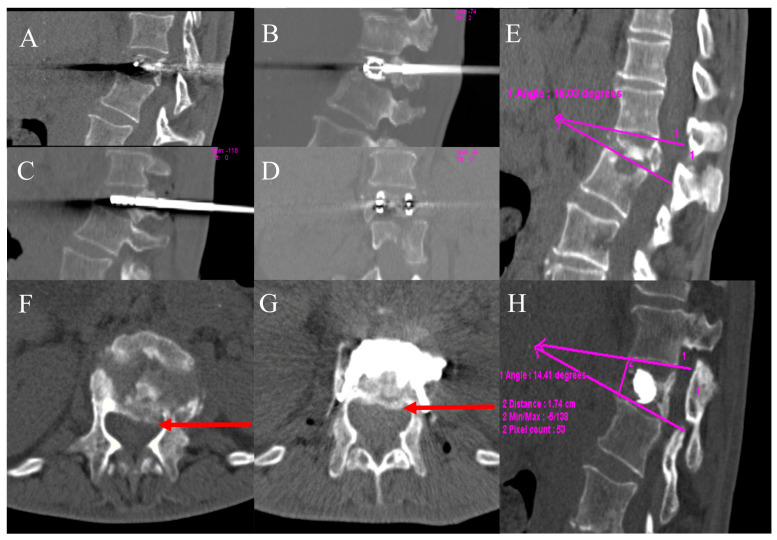
A 68-year-old male with L1 metastasis from colon cancer. The severe vertebral collapse of the L1 with retropulsion (arrows in axial (**F**) and sagittal views, (**A**)). After PVR treatment (axial, (**G**) coronal, (**D**), and sagittal views, (**B**,**C**), vertebral body height, posterior somatic wall realignment, and vertebral canal width recovery were obtained (axial, (**G**) and sagittal views, (**H**)). The angle of kyphosis resulting in less dislocation of bone fragments in the spinal canal, and the vertebral height was restored (angle (lines 1-1) 16° before treatment (**E**) vs. 14° after treatment (**H**)).

The restoration of the vertebral body affected by pathological collapse with a minimally invasive percutaneous approach is the main innovative aspect of the technique and, although a limited sample, the procedure allows recovery of height, even of severe pathological vertebral collapse, as shown in Figure 7.

The main novelty of the method is based on the introduction of the combined ablative and VTP treatment of the intermediate step of the vertebral SJ implant in order to recover the vertebral morphology, realign the column loading curve, and to resolve the posterior compression phenomena caused by the disease’s epidural extension. In our sample of treated patients, during the imaging follow-up, it was found that the recovery of the height of the anterior wall and the middle third of the vertebral body led to a realignment of the posterior wall as well. The recovery of both vertebral height and morphology results in a reduction of the extra-vertebral extension and epidural compressive phenomena [34].

The technical success is also associated with the improvement of two clinical aspects related to vertebral collapse. First, a recovery of bone height and consistency guarantee both analgesic and functional improvement, the gaining of pain control, and recovery of lost mobility.

Second a local tumor control thanks to the combined use of ablative techniques and cementoplasty. In our experience, the probability of VPT-related fracture of the treated and distant vertebrae is also reduced, and the cement distribution is improved, reducing procedural leaks.

In the small cohort examined, we obtained encouraging results in terms of the technical success, analgesic response, and recovery of the patients’ mobility. Furthermore, no residue or local recurrence was identified in the treated sites during follow-up. Therefore, we believe that this innovative procedure PVR could represent a further treatment option for advanced vertebral involvement when severe bone collapse and extra-vertebral extension limit safe vertebroplasty.

This study has some limitations, such as the small sample size, single-center study, retrospective design, no control group, and short duration of follow-up.

Despite these limitations, PVR represents a promising new approach in the minimally invasive landscape of spine metastases treatment, combining pain reduction, increased spine stability, and local tumor control.

## 5. Conclusions

This preliminary result suggests that a combined treatment of microwave ablation and bilateral expandable titanium SpineJack implants followed by vertebroplasty is a safe and effective procedure for the treatment of pathological compressive vertebral fractures.

The vertebral stabilization achieved allows early and persistent pain relief, increasing patient mobility, improving recovery of walking capacity, and providing local tumor control. 

## Figures and Tables

**Table 1:** Population characteristics and procedure details.

Patient	Age	Gender	Primary Cancer	Treated Vertebrae	Total n° SJ Impalnts	Total n° MWA	Complications
1	71	F	breast	L5	2	1	
2	69	F	breast	L1	2	1	
3	56	M	thyroid	L1	2	2	
4	64	M	lung	L3, T12	4	1	T12 vertebra fracture 7 days after L3 treatment
5	55	M	oral cavity	T10	2	1	
6	58	M	colon	L3, T12	2	2	
7	67	M	lung	L2	2	1	
8	66	M	NET	L3, T12	2	2	
9	49	M	kidney	L2	2	2	intradiscal leakage
10	72	F	breast	L1	2	1	
11	56	M	testicle	T12	2	1	
12	80	F	pancreas	L1, T12	4	4	
13	71	M	lung	T7	2	2	
14	76	F	kidney	T10, T12	3	4	
15	69	M	lung	T9, T10, T11	6	2	T11 vertebra fracture 7 days after T9–T10 treatment
16	66	F	breast	L5	2	1	
17	64	F	oral cavity	L1	2	1	
18	74	M	lung	L1, T12	4	4	
19	68	F	breast	L1, L2, L3	6	3	
20	75	F	parotid	L32	2	1	
21	62	F	breast	L1	2	2	anterolateral cement leakage
22	51	F	breast	L1	2	1	
23	72	F	thyroid	L1	2	1	
24	75	F	breast	T12	2	1	posterolateral cement leakage
25	41	M	melanoma	T10	2	2	
26	73	M	bladder	T8, T9	4	2	
27	76	F	breast	T11	2	1	
28	72	F	lung	T6, T7	4	2	

## Data Availability

Data are available on request.
